# Centrifuge modeling of rocking-isolated inelastic RC bridge piers

**DOI:** 10.1002/eqe.2451

**Published:** 2014-07-07

**Authors:** Marianna Loli, Jonathan A Knappett, Michael J Brown, Ioannis Anastasopoulos, George Gazetas

**Affiliations:** 1 School of Civil Engineering, National Technical University of AthensGreece; 2 Division of Civil Engineering, University of DundeeUK

**Keywords:** centrifuge modeling, seismic performance, soil–structure interaction, rocking isolation, capacity design, concrete failure

## Abstract

Experimental proof is provided of an unconventional seismic design concept, which is based on deliberately underdesigning shallow foundations to promote intense rocking oscillations and thereby to dramatically improve the seismic resilience of structures. Termed *rocking isolation*, this new seismic design philosophy is investigated through a series of dynamic centrifuge experiments on properly scaled models of a modern reinforced concrete (RC) bridge pier. The experimental method reproduces the nonlinear and inelastic response of both the soil-footing interface and the structure. To this end, a novel scale model RC (1:50 scale) that simulates reasonably well the elastic response and the failure of prototype RC elements is utilized, along with realistic representation of the soil behavior in a geotechnical centrifuge. A variety of seismic ground motions are considered as excitations. They result in consistent demonstrably beneficial performance of the rocking-isolated pier in comparison with the one designed conventionally. Seismic demand is reduced in terms of both inertial load and deck drift. Furthermore, foundation uplifting has a self-centering potential, whereas soil yielding is shown to provide a particularly effective energy dissipation mechanism, exhibiting significant resistance to cumulative damage. Thanks to such mechanisms, the rocking pier survived, with no signs of structural distress, a deleterious sequence of seismic motions that caused collapse of the conventionally designed pier. © 2014 The Authors *Earthquake Engineering & Structural Dynamics* Published by John Wiley & Sons Ltd.

## 1. Background and Objectives

Capacity design, which forms the cornerstone of modern seismic design, aims at controlling seismic damage by strategically directing inelastic deformation to structural components, which are less important to the overall system stability 1. Although this design approach is enforced or at least encouraged by modern seismic codes, it is conventionally limited to the superstructure. The foundation system is contrastingly treated conservatively. Specifically, the current foundation design leads to a strong unyielding foundation soil system by adopting overstrength factors to ensure that their ultimate capacity is reliably greater than the largest moment to be transmitted by the pier column.

Field evidence suggests that although new structures, complying with this capacity design rationale, are generally safer than the older ones, they remain vulnerable to very strong shaking. In fact, an alarmingly large number of modern structures have suffered intense damage leading to partial or total failure in recent earthquakes [e.g., 2–3] signaling the need to rethink the effectiveness of the aforementioned design practice. In response, a number of studies have explored the possibilities and constraints of an alternative design concept: allowing the development of ‘plastic hinging’ in the soil or at the soil–foundation interface, so as to reduce the possibility of damage to the elements of the structure.

Focusing on surface foundations, where nonlinearity manifests itself through uplifting and/or soil yielding, a ‘reversal’ of the current capacity design principle is proposed: the foundation is intentionally underdesigned under the seismic actions compared with the supported column to promote rocking response and accumulation of plastic deformation at the soil foundation interface. Supporting evidence for this new approach has been provided by the following theoretical and empirical findings:

- Several theoretical and numerical studies on the rocking response of rigid blocks and elastic single-degree-of-freedom (SDOF) oscillators [e.g., 4–7] provide compelling evidence that uplifting drastically reduces the inertial load transmitted into the oscillating structure.
- Because of the transient and kinematic nature of seismic loading, rocking response does not lead to overturning even in the case of very slender structures 8–14, except in rather extreme cases of little practical concern.
- Referred to as rocking isolation, allowing for foundation uplift has been proposed, and in a few exceptional cases employed in practice, as a means of seismic isolation 15–17.
- Even in the case of relatively heavily loaded footings or footings on soft soils, when rocking is accompanied with yielding of the supporting soil (and possibly momentary mobilization of bearing capacity failure mechanisms), substantial energy is dissipated in the foundation providing increased safety margins against overturning owing to the inherently self-centering characteristics and the ductile nature of rocking on compliant soil [e.g., 18–23].
- Most importantly, a number of studies have recently investigated the scheme of rocking isolation, with emphasis on its effects on structures, which consistently point to a beneficial role of nonlinear foundation behavior for the overall system performance. Previous studies include the numerical and experimental work of 24–26 in the domain of framed building structures, as well as those of 27–30 in the domain of bridges.
- A variety of modern numerical tools have been developed enabling comprehensive modeling of nonlinear rocking response 31–37, alleviating to some degree the skepticism regarding the uncertainties traditionally associated with prediction of the performance of rocking foundations for use in design.

On the basis of the exploratory work of 27, this study seeks to provide experimental verification of their numerical findings suggesting that although a conventionally designed reinforced concrete (RC) pier on an adequately large shallow foundation would suffer structural failure of the RC column and collapse in an earthquake sufficiently exceeding its design limits, rocking motion of an alternative underdesigned foundation would allow the same pier to survive even extreme shaking scenarios. To this end, it was necessary to realistically model the various attributes of nonlinear response of both the structural element (RC column) and the soil foundation interface, therefore necessitating the use of the following:

(1) A new scale model reinforced concrete 38 capable of replicating stiffness, strength, failure mode, and post-failure response of the bridge pier.
(2) The University of Dundee (UoD) centrifuge earthquake simulator (EQS) to accurately replicate nonlinear soil behavior and provide repeatable replication of historically recorded earthquake motions.

A series of centrifuge tests were performed to investigate and compare the performance of the two RC model bridge piers, having the same structural section in each case, but each representing one of the two considered design approaches, namely, a conventional design and a rocking isolation design. Presented in this paper are the results from four of these tests involving a variety of shaking scenarios using real historical ground motions. Previous studies have simulated similar problems in the centrifuge by introducing reduced structural model cross sections (mechanical fuses) to control the locations of inelastic deformation and strength within structures usually made of aluminum [e.g., 29, 39]. Despite the valuable insights of such testing, there are a number of unavoidable limitations:

(1) The location of any plastic damage must be defined a priori and any moment redistribution within the section is therefore suppressed.
(2) The axial load–moment capacity interaction behavior for aluminum is not of the same shape as for an RC section (in which axial load initially increases the moment capacity); therefore, any axial load redistribution within the section will not be captured correctly.
(3) The response of a fuse (whether in the form of notches or of artificial hinges) will not show the degradation in the moment–curvature behavior typical of reinforced concrete sections under cyclic loading.

The tests presented herein therefore attempt to model the inelastic response of RC elements in the centrifuge more realistically, namely, by using a new scaled model reinforced concrete consisting of a geometrically scaled steel reinforcing cage embedded within a cementitious material, while simulating the entire soil–foundation–structure system as a whole. In this way, the correct foundation behavior can be modeled simultaneously with a structure having fidelity of response closer to that of a larger scale structural element test than has been achievable to date. Hence, the paper pursues two additional objectives: (i) presentation of the design and construction of realistically scaled RC model piers for use in centrifuge experiments and (ii) a report of 1–g calibration testing to verify the bending behavior of these piers. In the following, properties and results are given at prototype scale, unless otherwise stated.

## 2. Design of the Pier–Foundation Systems

**Figure 1:**
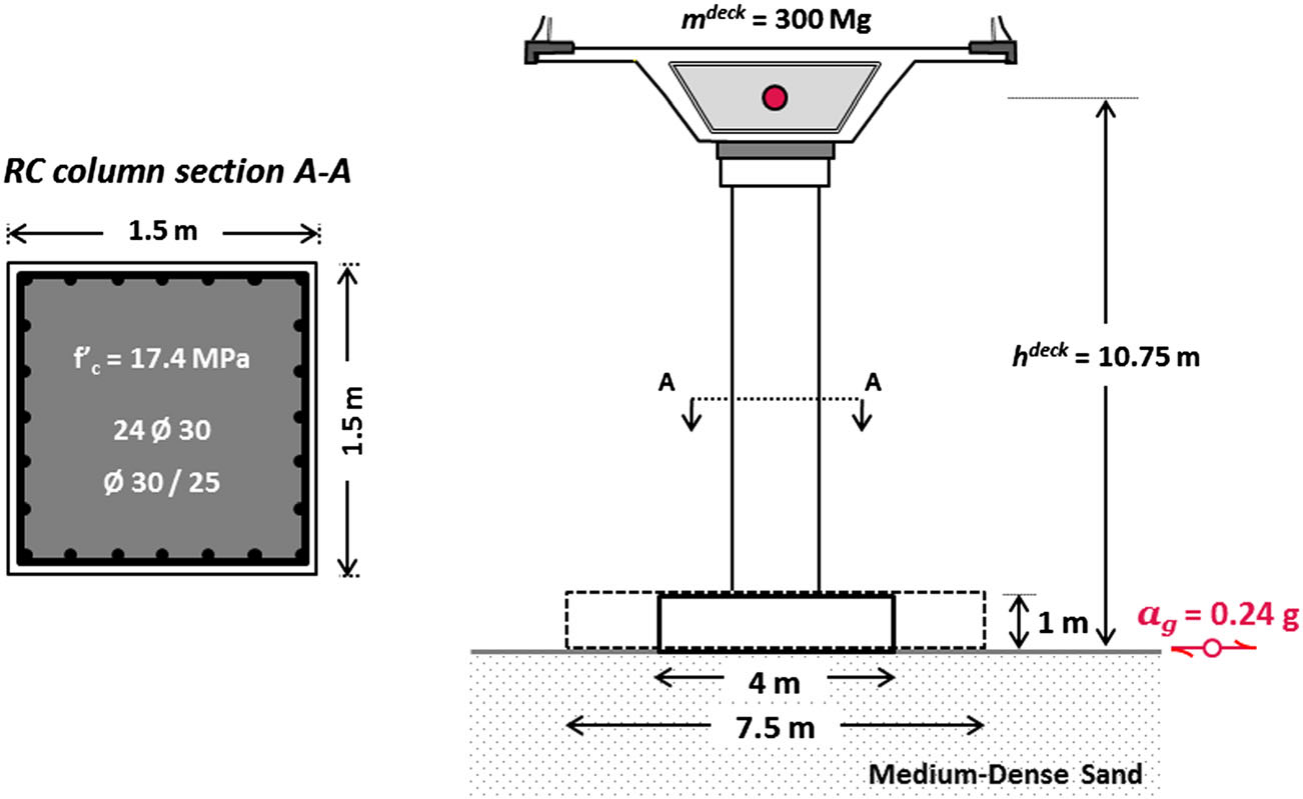
Schematic definition of the studied problem.

Figure 1 illustrates the conceptual prototype of a modern bridge pier designed in accordance with Eurocode specifications for RC structures and seismic actions 40,41. The deck is a cast-in-place concrete box girder with a total dead load *q_l_* = 200 kN/m, free to rotate oscillating in the transverse direction. Thus, a 10.75 m tall (to the center of mass of the deck, including the footing) cantilever is considered carrying the concentrated mass of the supported deck (*m^deck^* = 300 Mg) and comprising a RC column with a cross section of 1.5 m × 1.5 m (cross-sectional area *A_c_* = 2.25 m^2^).

The RC column was designed to withstand static loads and seismic actions due to a design ground acceleration *α_g_* = 0.24 g. Using the EC8 specified acceleration response spectrum for a Type C soil profile and assuming ductile behavior (*q* = 3), the design spectral acceleration may be calculated as *S_A_* = 0.23 g. Considering the availability of typical model reinforcement sizes and properties (discussed in the following section) as well as reasonable geometries to allow ease of construction, it was deduced that uniformly spaced longitudinal reinforcement of 24 bars of *d_bl_* = 30-mm diameter combined with shear links of the same diameter spaced at 250 mm was required to effectively carry design loads, using a concrete of compressive strength *f_c_* = 17.4 MPa (cylinder strength). Table 1 summarizes calculations regarding design loads (with reference to the base of the column) and verification of adequate reinforcement in shearing and bending (moment capacity and ductility). It should be noted that bending response was predicted through cross-sectional analysis employing the USC_RC software 42.

**Table 1:** RC column load calculations and design assessment with respect to the Eurocode.

Loading and Resistance of RC section		
Axial load	N:MN	3.426
Shear load	V:MN	0.677
Moment load	M:MNm	6.600
Normalized axial load	*n* (=N/A_c_f_c_)	0.088
Normalized shear load	*ν* (=V/A_c_f_c_)	0.017
Normalized moment load	m (=M/bA_c_f_c_)	0.112
Moment capacity	M_u_:MNm	6.816
Factor of safety	FS	1.1
Curvature ductility	μ_φ_	18
Displacement ductility	μ_Δ_	6.5
Capacity design shear	V_Ed_:MN	1.022
Shear reinf. yield shear force	V_Rd,s_:MN	3.415
Maximum member shear force	V_Rd,max_:MN	10.257
Shear resistance	V_Rd_:MN	3.415
Factor of Safety	FS	3.3

RC, reinforced concrete.

The pier is founded on a shallow, 10-m thick, layer of medium density sand with a square (*B* × *B*) footing. Two different footing dimensions are studied, representing the two design alternatives. Table 2 summarizes the two foundation designs listing: static and seismic loads (*Q_E_*, *M_E_*), design seismic actions (*Q_Ed_*, *M_Ed_*), and ultimate shear and moment capacities (*Q_u_*, *M_u_*) and the factors of safety for static vertical loads (*FS_V_*) and seismic loads (*FS_E_*). It should be noted that, apart from the small difference in the total vertical load *N_tot_* (resulting from the different foundation sizes), the two piers are subjected to the same design loads (as they are identical in geometry) and to the same design ground acceleration *α_g_*. *Q_Ed_* and *M_Ed_* are calculated according to the capacity requirements for the foundation overstrength, which calls for seismic design actions on the foundation be substantially magnified (by as much as 40% in the case of a cantilever structure) in comparison with the actual seismic loads at the column base. Yet, only the conventional foundation complies with this requirement (hence having *FS_E_* > 1, as opposed to the rocking foundation which has *FS_E_* < 1). Combined with the limitations on the maximum allowable eccentricity ratio (*e* = *M*/*N* < 2*B*/3), this overstrength requirement leads to the conventional foundation being significantly larger (*B* = 7.5 m) than the rocking-isolated one (*B* = 4 m). Foundation capacity was, in a first step, calculated using the well-established failure envelope relationship of 43

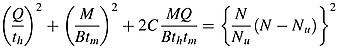
1where *t_h_*, *t_m_*, and *C* are parameters taken equal to 0.52, 0.35, and 0.22, respectively (determined through curve fitting of experimental results), and *N_u_* is the ultimate capacity in pure vertical loading. In addition to theoretical predictions, the foundation capacities were also calculated using numerical simulations with finite elements (FE)—details are reported in 44.

**Table 2:** Foundation design: summary of actions and factors of safety (FS).

Property	Unit	Conventional	Rocking
Breadth	B:m	7.5	4
Total vertical load	N_tot_:MN	4.9	4
Seismic shear load	Q_E_:MN	0.7	0.7
Seismic moment load	M_E_:MNm	7.3	7.3
Design shear action	Q_Ed_:MN	0.7	0.7
Design moment action	M_Ed_:MNm	7.3	7.3
Ultimate shear capacity	Q_u_:MN	1.2	0.5
Ultimate moment capacity	M_u_:MNm	12.9	4.8
Factor of safety in vertical loading	FS_V_	18	3.5
Factor of safety in combined (seismic) loading	FS_E_	1.77	0.66

## 3. Experimental Methods

The experimental program was carried out at the UoD and involved three different parts. First, eight RC model columns were tested in the standard four-point bending to optimize their construction procedure and verify their bending response. Second, two dynamic centrifuge model tests were carried out using elastic (aluminum) piers to measure the ultimate shear moment capacity (*Q_u_ M_u_*) in the combined *N–Q–M* loading space for the two alternative foundations and check their *M–θ* response in comparison with FE predictions. In lieu of static pushover tests, this was achieved by exciting the soil–structure model with Ricker wavelets of suitable characteristics (*PGA* = 0.6 g and dominant frequency *f_E_* = 1 Hz) causing the foundations to respond well beyond their nonlinear regime. Close approximation of the monotonic *M–θ* backbone curve was achieved, and the results were in good agreement with FE predictions. These characterization tests are reported in 44. The third element of testing, which is the main scope of this paper, involved four centrifuge model tests, two for each of the alternative designs, wherein the RC model piers were subjected to different earthquake scenarios using real records of varying intensities. All tests were conducted at a scale of 1:50 (*n* = 50) and at 50 g. The following section describes the modeling and testing procedures.

### 3.1. Reinforced concrete column scaled models: construction and validation

Modeling cementitious material at reduced scale is prone to size effects, owing to the presence of aggregates, and can lead to significant overstrength when the scale reduces substantially (*n* > 10), as is usually the case in geotechnical centrifuge model testing. Therefore, this study has employed a novel scale model reinforced concrete, developed and validated by Knappett and co-workers 38,45,46, which realistically scales down both material stiffness and strength and reasonably replicates the response of RC structural elements under bending and shear loads. A gypsum-based mortar (beta-form surgical plaster) was used as a cementitious binder. Uniformly graded Congleton HST 95 silica sand 47 served well in modeling the aggregate phase of the concrete because its particle size distribution reasonably approximates the geometrically scaled grading curve of typical coarse aggregate at 1:50 scale. The plaster (P) and sand (S) were mixed with water (W) at a ratio of P/S/W = 1:1:1 by weight. The compressive strength of the produced concrete model was measured through tests on 100 × 100 mm standard cubes as *f*_*c*,*100*_ = 26.3 MPa (equivalent to cylinder strength of *f*'*_c_* = 17.4 MPa).

**Figure 2:**
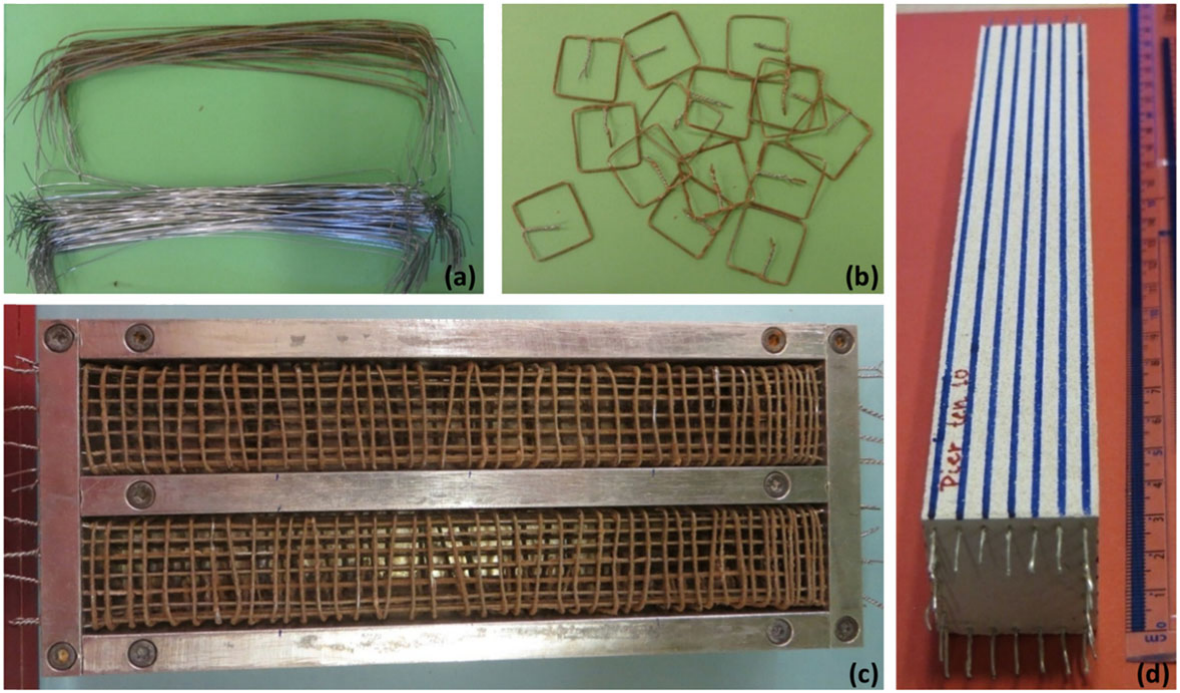
Construction of reinforced concrete model columns: (a) longitudinal steel with (upper) and without (lower) sand coating; (b) shear links; (c) steel within formwork; and (d) column model.

Reinforcement was modeled by stainless steel wire with a measured yield stress of *f_y_* = 460 MPa (at 0.2% permanent strain), whereas the post-yield stress–strain response indicated significant hardening, making it closely representative of high yield reinforcement. The wire was cut and bent appropriately to produce scaled longitudinal bars (Figure 2(a)) and confining transverse reinforcement (Figure 2(b)). For ease of construction, wire of the same size, 0.6 mm in diameter at model scale (30-mm diameter at prototype scale), was utilized for both longitudinal and shear reinforcement. It should be noted that using shear links of smaller diameter, as is usually the case in practice, would require spacing lengths smaller than 5 mm at model scale to achieve the desired ductility, which would significantly hinder model fabrication. For both types of reinforcement, the wire was coated with HST95 silica sand using a fast-drying epoxy resin to produce a realistically rough interface between the steel and the model concrete.

Fabrication of the reinforcement assembly was challenging because of the scale of the produced columns. The 200-mm long column model contained a total length of more than 5 m of wire modeling longitudinal reinforcement and 45 shear links uniformly spaced at a distance of approximately 5 mm. Anchoring of longitudinal reinforcement was achieved by providing an additional length of about 10 mm on each side of the column, which was bent and fixed within the foundation or the deck plates. Test units were cast in a custom-built formwork (Figure 2(c)), which allowed casting of two columns at a time. The column models (Figure 2(d)) were left to cure for at least 2 weeks before testing.

A total of 19 columns were produced, most of which were used in bending tests intended to verify the moment–curvature response of the column section and the capacity of the column–foundation joint. It is important to note that in addition to shear links, the alternative of using continuous spiral shear reinforcement was also investigated but was found less effective in producing sections with a constant core area resulting in less accurate replication of the cross-sectional capacity.

**Figure 3:**
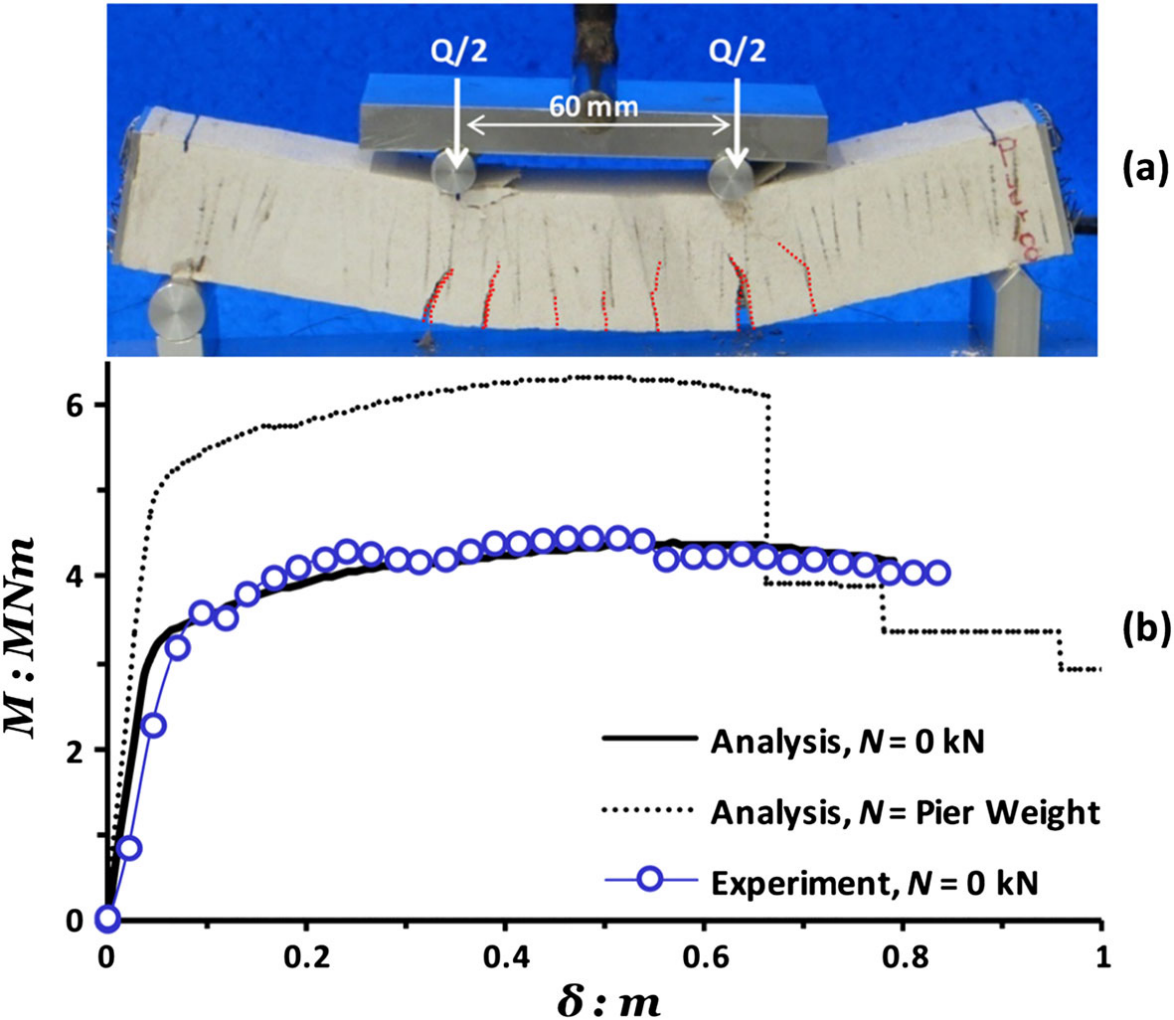
Bending response of RC column: (a) photograph of a four-point bending test on a model column; (b) comparison of computed monotonic moment–deflection response with bending test results in the absence of axial load (*N* = 0 MN) and analytical prediction of overstrength due to the pier dead loads (*N* = 3.4 MN).

A series of standard four-point bending tests were carried out under displacement control. Figure 3(a) shows a typical test on a column model identical to those used in the centrifuge, highlighting the loading arrangement and the typical mode of failure. It can be seen that the observed crack pattern is typical of a column designed to fail in flexure (i.e., containing sufficient shear reinforcement to suppress shear failure). This was expected considering the calculated moment and shear capacities of the section given in Table 1. Vertical cracks may be identified within the 60-mm wide central span, which is subjected to pure bending (constant moment) while their inclination evidently changes in the two zones of combined moment shear loading between the load points and supports. Figure 3(b) compares the measured bending behavior of the column section (in prototype scale), in terms of bending moment (*M*) versus deflection (*δ*), with numerical predictions using USC-RC for pure bending conditions (without axial load) indicating very satisfactory agreement. Unfortunately, the available laboratory equipment did not allow testing with axial load to measure the effect of the pier weight on the section response. Yet, the *M δ* response of the concrete section under axial load equal to the pier weight (*N* = 3.4 MN) was calculated numerically and is also shown in Figure 3(b). As expected, the weight of the pier, which is present in the centrifuge model tests, results in considerable increase of the section moment capacity, although at the cost of reduced ductility. It is important to note that the USC-RC-predicted moment capacity for the actual axial load is in good agreement with the maximum moment load recorded in the centrifuge tests.

**Figure 4:**
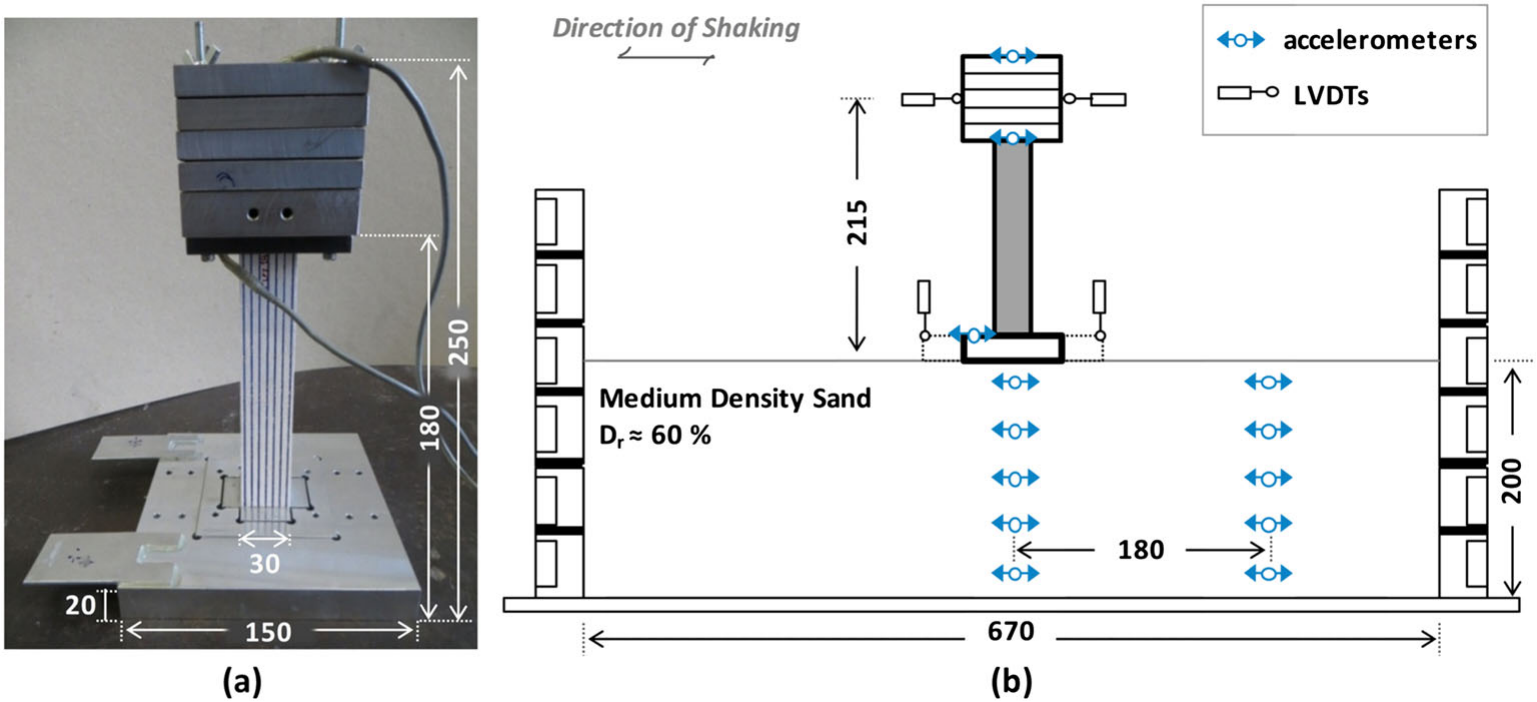
Experimental setup:(a) photo of the pier model assembly (mass, RC column, and foundation) and attached accelerometers indicating characteristic dimensions and (b) schematic cross section of the model within the laminar box showing instrumentation. All dimensions are in millimeters (model scale).

Only the column, where structural failure was expected to take place, was modeled using the RC material, whereas the deck and the foundation were made of steel and aluminum, respectively. Figure 4(a) displays a photo of the mass-column foundation assembly indicating key dimensions. Care was taken in the design and setup of the deck-to-column and column-to-foundation joints to achieve full fixity. The elastic fixed-base vibration period of the pier models was measured through free vibration tests as *T_0_* = 0.24 s (in prototype scale), which presumably does not account for foundation geometry, being therefore the same for both design alternatives. Yet, soil structure interaction is expected to drastically increase this value especially in the case of the rocking pier.

### 3.2. Centrifuge modeling

Similitude is an important consideration in physical modeling using reduced scale models that are intended to capture the response of field-scale prototypes. Centrifuge modeling is particularly useful for the investigation of soil–foundation–structure interaction problems where realistic simulation of the stress dependent soil behavior plays a key role. Thanks to the enhanced gravity field in a geotechnical centrifuge, a 1:*n* scale model will have the same effective stress acting at homologous points in the model soil and the full-scale prototype soil. A set of scaling laws have been developed to achieve similitude in centrifuge modeling, as detailed by 48,49.

Dynamic centrifuge model testing was carried out in the 3.5-m diameter beam centrifuge of the UoD. A schematic cross section of the model including key dimensions and instrumentation is shown in Figure 4(b). The 200-mm deep soil layer was prepared by air pluviation of dry fine Congleton silica sand (HST95, *γ*_max_ = 1758 kg/m^3^, *γ*_min_ = 1459 kg/m^3^, *D*_60_ = 0.14 mm, and *D*_10_ = 0.10 mm) to achieve a uniform relative density *D*_r_ = 60%. The soil model was prepared within an equivalent shear beam (ESB) container with flexible walls, described in 50. It was instrumented using two vertical arrays of five ADXL78 MEMS accelerometers, one array buried under the foundation centerline and the other at a distance large enough to record free field response. The motion of the pier was recorded using identical accelerometers attached to the foundation and the deck. Vertical displacements of two foundation corners, recorded by Linear Variable Differential Transformers (LVDTs), were used to calculate settlement and rotation in the direction of shaking. Another pair of LVDTs recorded the horizontal displacements of the deck center of mass.

### 3.3. Testing protocol

**Figure 5:**
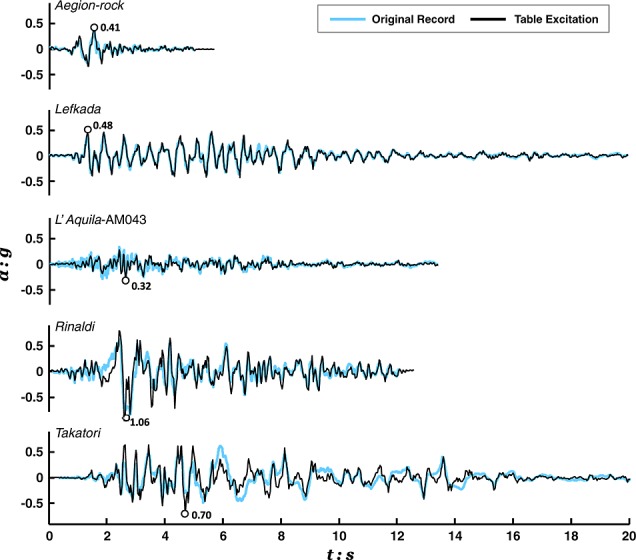
Shaking table acceleration time histories used in the different earthquake scenarios, compared with target records from earthquakes of different magnitudes in Greece, Italy, USA, and Japan.

Ground motions were applied using an Actidyn Q67-2 servo-hydraulic EQS. Taking advantage of the effectiveness of this device in simulating desired motions 51, an ensemble of records from historic earthquakes were utilized as base excitation. The motions were band pass filtered between 0.8 and 8 Hz to match the controllable frequency range of the EQS and then calibrated during a preliminary test on a ‘dummy’ model to allow highly repeatable reproduction of original records with reasonable accuracy, as illustrated by comparison of achieved and target acceleration time histories in Figure 5.

**Figure 6:**
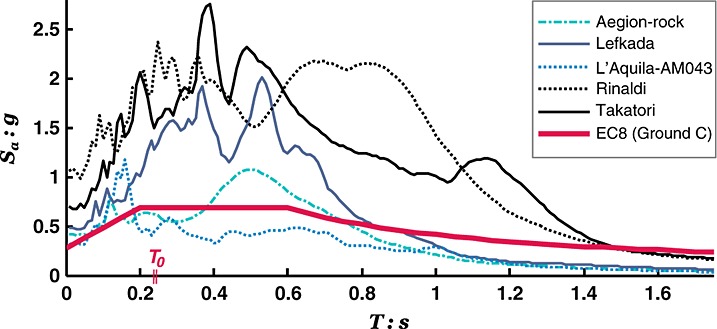
Acceleration response spectra (*ξ* = 5%) of the shaking table excitations compared with the EC8 design spectrum for ground Type C and *a_g_* = 0.24 g.

Figure 6 shows the 5% damped acceleration response spectra of the actual shaking table motions and compares them to the EC8 design spectrum. Evidently, the Aegion-rock and L'Aquila-AM043 motions, recorded during the 1995 *M_s_* 6.2 Aegion (Greece) and the 2009 *M_s_* 6.3 L'Aquila (Italy) earthquakes, are typical medium intensity excitations of the order of magnitude assumed in design. By contrast, the Rinaldi(228) and Takatori(000) motions, recorded during the 1994 *M_s_*6.8 Northridge (USA) and 1995 *M_JMA_* 7.2 Kobe (Japan) earthquakes, are very strong motions that dramatically exceed the design level. Characterized by near fault directivity effects, these latter motions have increased spectral ordinates within a particularly broad band of periods (especially in the range of particular interest, i.e., *T* ≥ *T_0_*). Having considerable duration and number of cycles, the record of the 2003 *M_s_* 6.4 Lefkada earthquake (Greece) represents an intermediately strong, yet still above design levels, seismic scenario.

Central to the issue of seismic performance evaluation is the recognition that damage in a component is cumulative, and the level of damage depends not only on the maximum deformation but also on the history of deformations. In practice, structures may, over their lifespan, be subjected to several motions of varying intensities. Thus, attempting to account for seismic history, the performance assessment undertaken here involves a succession of motions considering two different seismic history scenarios. In *Earthquake Scenario A*, the ground motion order was such that the intensity roughly increased throughout the test, as if a number of weak or medium intensity motions were to precede a major catastrophic event, either in a sense of foreshocks or as independent events taking place in a relatively short period. *Earthquake Scenario B* explores the case where a very strong earthquake is followed by weaker shaking events or aftershocks. The two scenarios were investigated independently for each of the two designs considered with the exact testing protocol as summarized in Table 3. It should be noted that in some cases, when scenarios *A* or *B* did not lead to collapse, additional shaking was applied using the Takatori record until ultimate or practical failure was reached.

**Table 3:** Testing program: sequence of seismic excitations.

Test	Pier design	Motion protocol						
1	Rocking isolation	A	Aegion	Lefkada	L'Aquila	Rinaldi	Takatori	—
2	Conventional design		Aegion	Lefkada	L'Aquila	—	—	—
3	Rocking isolation	B	Rinaldi	Aegion	Aegion	L'Aquila	Takatori	Takatori
4	Conventional design		Rinaldi	Aegion	Aegion	L'Aquila	Takatori	—

## 4. Experimental Results

In this section, the seismic performance of the two pier–foundation systems during seismic scenarios *A* and *B* is evaluated and systematically compared. Focusing on damage accumulation due to successive earthquakes and the importance of shaking history, time histories of recorded illustrative demand parameters, such as deck acceleration and displacement, as well as moment-rotation and shear force-displacement hysteretic response loops are subsequently presented at prototype scale.

### 4.1. Response to Earthquake Scenario A

**Figure 7:**
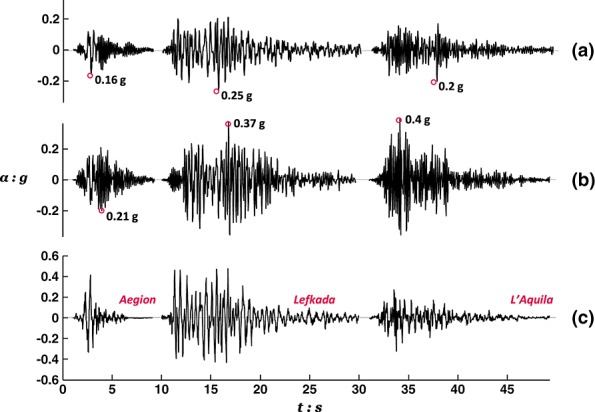
Acceleration time history sequence recorded during Earthquake Scenario A at (a) the deck of the rocking pier; (b) the deck of the conventional pier; and (c) the model base.

Figure 7(a,b) shows the sequence of acceleration time histories recorded at the center of mass of the deck (the average of measurements at the top and bottom of the deck mass) in each of the two alternative designs during shaking with the first three motions of Earthquake Scenario A (plotted in Figure 7(c)). As anticipated, the rocking pier experiences invariably lower acceleration than the conventional pier. This advantage, known as rocking isolation, is the result of the difference in the ultimate moment capacity of the two designs (Table 2): the ultimate moment capacity of the RC column section is larger than the ultimate moment capacity of the rocking foundation. This advantage becomes more significant as excitation intensity increases and the ultimate capacities are mobilized. Hence, upon shaking with the L'Aquila record, the maximum transient demand experienced by the rocking pier is half the demand on the conventional pier.

The rocking isolation effect can be easily quantified with regard to the moment capacity of the rocking foundation, being therefore potentially useful for the estimation of seismic demand in design. Assuming pure rotational movement about the footing midpoint, equilibrium requires the critical value of the maximum acceleration developed in the deck (*α_c_*) to depend on the ultimate moment capacity of the foundation (*M_u_*): *α_c_* = *M_u_*/*m^deck^h*. Given the theoretical capacity of the rocking foundation (Table 2), *a_c_* may be calculated as 0.16 g. Yet, this value coincides with the measured peak mass acceleration only in the very first shaking event using the Aegion record. Thereupon, overstrength effects, associated with soil densification during shaking, lead to some considerable increase in this value, which yet remains substantially lower than the peak demand on the conventional pier. It should be noted that deviations of the maximum deck acceleration from the theoretical *α_c_* value may be also because of uncertainties in the estimation of *M_u_* and the simplifying presumption of pure rotational movement. Nevertheless, such overstrength effects, which lead to some considerable increase of *M_u_* and *α_c_* due to preceding loading cycles, have been identified in the past experimental studies and documented in 23,30.

**Figure 8:**
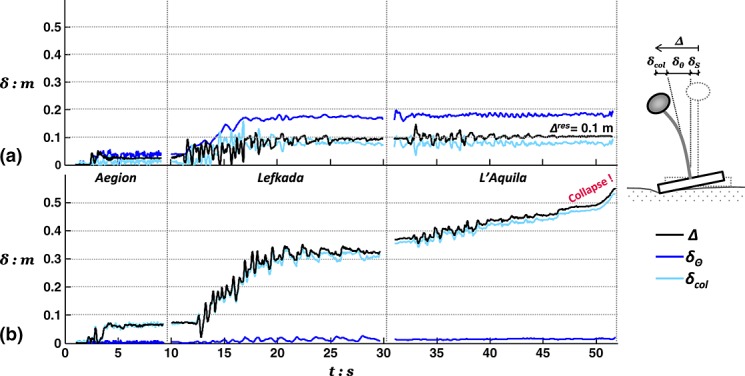
Total deck drift *Δ* of (a) the rocking pier and (b) the conventional pier, shown as the components of rotational movement *δ_Θ_* and flexural deformation *δ_col_* during shaking with Earthquake Scenario A.

On the basis of the aforementioned discussion, it may be deduced that even the relatively low intensity Aegion excitation is sufficient to momentarily mobilize the capacity of the rocking footing and induce its rocking–uplifting response. Indeed, as indicated by the deck displacement time history plots in Figure 8(a), the pier response is primarily controlled by rotational movement due to foundation rocking. Horizontal deck displacement measurements (Figure 8) are separated into their two main components: the lateral displacement due to foundation rotation (*δ_Θ_*) and due to the flexural deformation of the pier (*δ_col_*). The evolution of *Δ* throughout shaking serves as an index of the two systems seismic performance. Moreover, superimposing the contribution of *δ_Θ_* and *δ_col_* is intended to illustrate the dominating mode of response. It should be noted that, unlike *Δ* and *δ_Θ_*, *δ_col_* was not measured directly but calculated with respect to the measurements of the other two as *δ_col_* = *Δ* − *δ_Θ_*, assuming that for such slender oscillators, foundation sliding (*δ_S_*) is minimal. Evidently, as anticipated, in the case of the rocking pier, rotational movement prevails throughout the entire shaking sequence. The opposite is the case with the conventional pier, where deck drift is almost exclusively associated with deformation and failure of the RC column. More importantly, the rocking pier demonstrates a crucial advantage over conventional design. Not only does it experience significantly lower drift in the first shaking event (Aegion) but it also retains an increasingly favorable performance during the following earthquakes of greater intensity and duration. In particular, having experienced the first two excitations, it is practically unaffected by the sustained shaking of the L'Aquila record, resulting in a small total drift of 0.1 m (≈0.1% drift ratio). In contrast, the conventional pier accumulates large flexural deformations leading to approximately three times the deck drift seen for the rocking pier after the second motion and eventually failing with the third motion, having acquired drift levels of more than 50 cm (or ≈ 0.5% drift ratio). Note that in this test, failure is assumed to take place when the deck mass hits the horizontal LVDT, which prevents further movement in the direction of shaking. Yet, the rate at which *Δ* is observed to increase just before hitting the instrument (Figure 8(b)) implies imminent collapse of the pier column.

**Figure 9:**
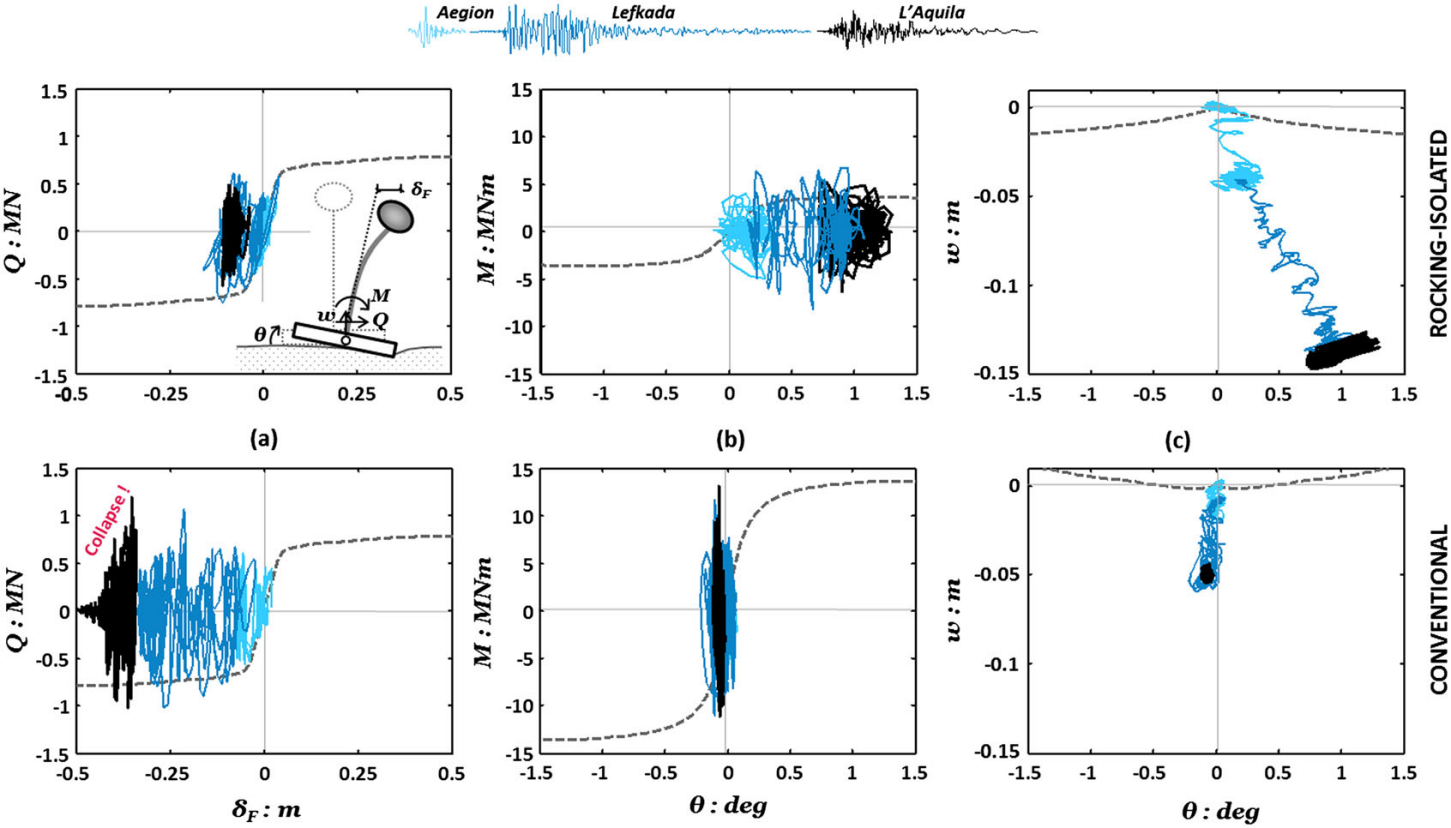
Rocking (top) versus conventional (bottom) pier hysteretic responses: (a) *Q–δ_col_* with reference to the base of the RC column; (b) foundation *M–θ*; and (c) foundation *w–θ* for Earthquake Scenario A.

Inelastic action is not only unavoidable but also essential in providing the required energy absorption to enable survival of the structure under intense seismic shaking. The two design alternatives, both relying on inelastic response, differ only in the component where the inelastic deformation is directed to. The adequacy of these nonlinear components (‘fuses’) may refer to their strength, ductility, cumulative damage resistance, and energy dissipation capacity. The effectiveness of the designated ‘fuses’, either the RC column section for conventional design or the soil–foundation interface for the rocking design, is of critical importance for the survivability of the system. Direct comparison of the two piers' ‘fuse’ response is highlighted by comparing the family of hysteresis plots depicted in Figure 9.

Figure 9(a) shows the response of the RC column during the three first earthquakes of Scenario A in terms of shear load (*Q* = *mα*) versus the flexural component of drift (*δ_col_*). It can be seen that the conventional pier column marginally mobilizes its lateral load capacity during the Aegion earthquake resulting in some rather minimal permanent flexural deflection (*δ_col_*/*h* < 0.1%). Subsequent loading with the stronger Lefkada motion causes an important number of excursions into the nonlinear regime, accumulating substantial permanent deformation at the column base. Nevertheless, designed according to modern code requirements, the pier column possesses adequate confinement and, hence, ductility to sustain such a significant number of loading cycles with no apparent deterioration of strength, yet at the cost of considerable permanent deflection *δ_col_* ≈ 30 cm (*δ_col_*/*h* ≈ 0.3%). However, having experienced this damage, the pier column appears unable to sustain further excitation with the equally strong L'Aquila motion, which exhausts its ductility capacity causing rapid deterioration of strength after a couple of cycles and eventually collapse. On the other hand, the column of the rocking pier responds, as expected, practically within the linear-elastic regime throughout the entire sequence.

Figure 9(b,c) summarizes the performance of the two foundations in the moment rotation and settlement rotation domains. Verifying its design, the conventional foundation responds linear elastically with increased rotational stiffness, in comparison with the monotonic backbone curve, owing to densification of the underlying soil. By contrast, the rocking foundation presents a broad moment–rotation hysteresis receiving comparatively larger rotational demand. Nevertheless, rotational movement is kept within tolerable margins, as may be judged with respect to the resulting deck deflections (Figure 8). Downwards movement prevails resulting in considerable settlement of the foundation from the first earthquake (≈5 cm, which is equal to the total settlement of the conventional foundation over the complete scenario), this being the main shortcoming of the rocking design. Foundation settlement increases drastically in response to the multiple shaking cycles of the Lefkada record, leading to a considerable amount of settlement (*w* ≈ 14 cm) by the end of the third earthquake. Nevertheless, despite the increased settlements, the rocking pier appears to have a crucial advantage over the conventional pier: not only does it avoid collapse but it also exhibits a particularly effective and ductile ‘fuse’ mode of response, as its rocking foundation sustains a large number of loading cycles with no apparent deterioration of strength.

Although the collapse of the conventional pier column after excitation with the L'Aquila record ended Test 2, Test 1 was continued by applying two additional very strong motions, the Rinaldi and the Takatori records. Figure 10 shows the time history response of the rocking pier during these last, particularly intense, excitations in terms of deck drift, foundation settlement, and deck acceleration.

**Figure:**
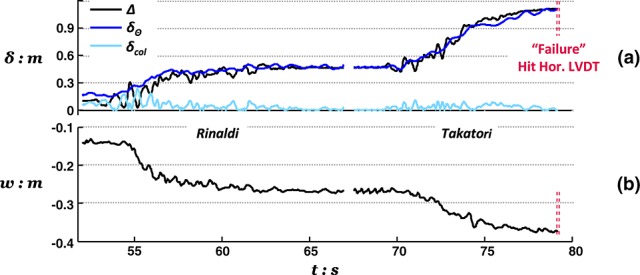
Response of the rocking pier subjected to successive base excitation with the Rinaldi and the Takatori motions, after having survived shaking with the three preceding lower magnitude motions of Earthquake Scenario A, in terms of (a) deck drift and (b) foundation settlement time histories.

**Figure 11:**
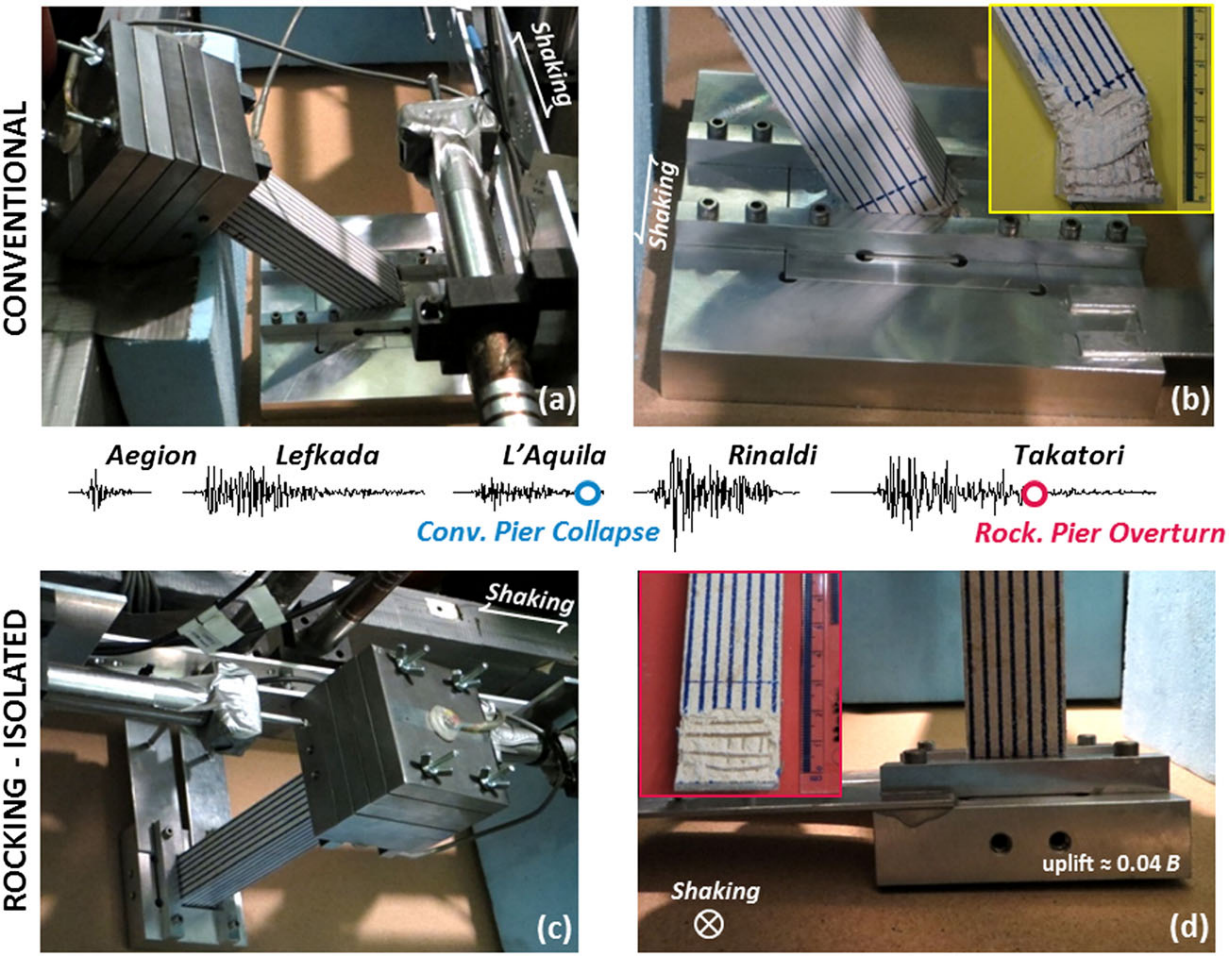
Photos of the bridge models after Tests 1 and 2 (Scenario A): (a) failure of the conventional pier after the first three medium-strong intensity motions and (b) the damage at the pier base compared with (c) the rocking pier subjected to the same motions plus two additional very strong motions, focusing on (d) foundation uplift.

Remarkably, despite having been subjected to a sequence of three earthquakes with intensity equivalent to, or exceeding, its design earthquake and having suffered considerable foundation deformation, the rocking pier survives the excess demands imposed by the Rinaldi motion. Displacements, in both horizontal and vertical directions, are naturally increased substantially (*w_res_* = 27 cm, *δ_tot_* = 45 cm), but the response is judged as satisfactory as the pier remains stable after such a deleterious sequence of earthquakes. The pier eventually failed (again by hitting the horizontal LVDT) during excitation with the last, extremely strong excitation using the Takatori record. Figure 11 shows images of the two models after testing, verifying the respective mode of response.

The realistic reproduction of flexural failure in the case of the conventional pier (Figure 1(b)) is worth observing, where the plastic hinge length is approximately equal to the column width, as expected in practice. Note, also, that after hitting the horizontal LVDT during the L'Aquila motion, the pier was found to have collapsed in the out-of-plane direction (Figure 1(a,b)). After being subjected to the entire sequence of Scenario A (five earthquakes), the rocking pier rotated significantly (Figure 1(c)) with evident foundation uplift, yet with its RC column remaining practically intact (Figure 1(d)).

### 4.2. Response to Earthquake Scenario B

**Figure 12:**
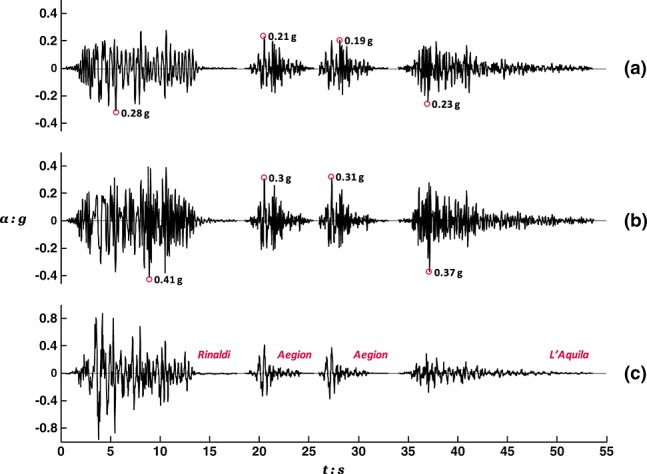
Acceleration time history sequence recorded during shaking with Earthquake Scenario B at (a) deck of rocking pier (smaller foundation); (b) deck of conventional pier (larger foundation); and (c) excitation.

The response of nonlinear systems strongly depends on the exact loading history. Hence, it was decided to further study the response of the two pier designs under an alternative earthquake sequence in order to generalize the previously made observations. This loading scenario differs from Earthquake Scenario A in that the very intense Rinaldi record is applied first, whereas the weaker Aegion and Lefkada records, subsequently imposed on the models, may be perceived as smaller aftershocks. Figure 12 shows the acceleration time history of the first four motions involved in this scenario (note that the Aegion record was applied twice) in comparison with the accelerations recorded at the decks of the two piers. Measurements are in qualitative agreement with those in Figure 7 verifying the beneficial effect of rocking isolation in drastically reducing seismic demands on the rocking pier.

**Figure 13:**
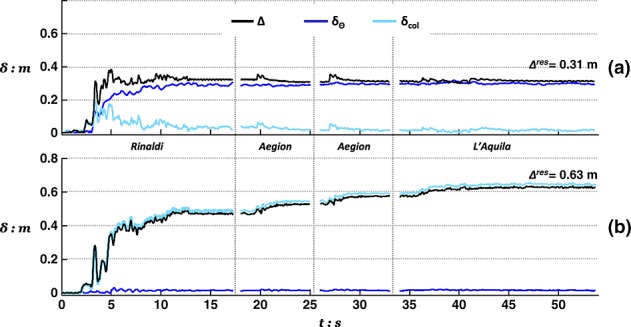
Total deck drift *Δ* of (a) rocking pier and (b) the conventional pier, shown as the components of rotational movement *δ_Θ_* and flexural deformation *δ_col_* during shaking with Earthquake Scenario B.

Figure 3 shows the evolution of deck drift for each of the pier models highlighting the contribution of rotational movement (*δ_Θ_*) and column deflection (*δ_col_*). Again, in agreement with results from Tests 1 and 2, it may be seen that for the rocking pier, deck deflections are mainly because of the foundation rotation, whereas column deflection plays a minor role. The opposite is the case for the conventional pier. The rocking pier suffers significantly less total drift than the conventional pier in the Rinaldi earthquake (31 cm, or *Δ*/*h* ≈ 0.3%, against 47 cm, or *Δ*/*h* ≈ 0.4%). Despite being considerably distressed by the first very strong motion, the rocking pier demonstrates a remarkably stable response during shaking with the following three smaller excitations. Showing surprising resistance against cumulative damage, the rocking foundation suffers little additional rotation and hence negligible additional deck displacement (Figure 13(a)). In contrast, each shaking event adds considerably to the deflection of the conventional pier (Figure 13(b)). By the end of Earthquake Scenario B, the conventional pier was subjected to twice the deck drift (≈63 cm) associated with the rocking alternative.

**Figure 14:**
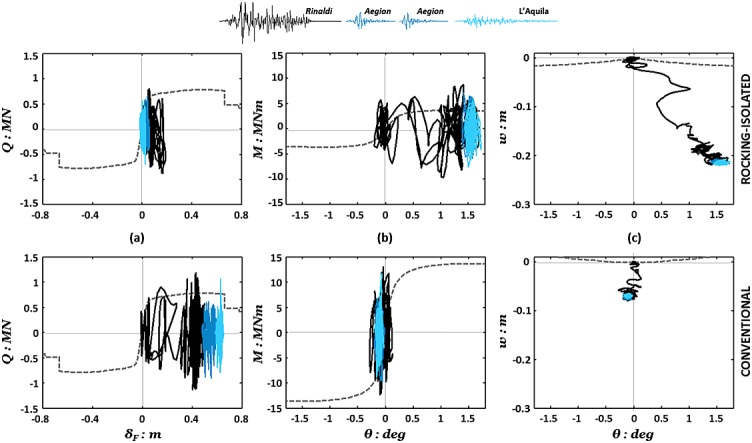
Rocking (top) versus conventional (bottom) pier hysteretic responses: (a) *Q–δ_col_* with reference to the base of the RC column; (b) foundation *M–θ*; and (c) foundation *w–θ* for Earthquake Scenario B.

Comparison of hysteretic responses recorded during the Rinaldi earthquake (Figure 4) shows strongly nonlinear behavior of the RC column or the foundation in the case of the conventional design or the rocking design, respectively. It is worth observing that this first strong motion pushes the response of the conventional column (Figure 4(a)) well within its nonlinear regime consuming more than half of its theoretical ductility capacity. The following lower magnitude earthquakes add up to the total ductility demand and the column is observed to be ‘on the verge of failure’ by the end of the shaking sequence, having exhausted its theoretical ductility margins. On the other hand, the rocking foundation exhibits good energy dissipating behavior with no deterioration of foundation capacity (Figure 14(b)). Furthermore, it is important to observe that, as with horizontal displacements, there is negligible additional settlement of the rocking foundation due to the post-Rinaldi earthquake loading (Figure 14(c)).

**Figure 15:**
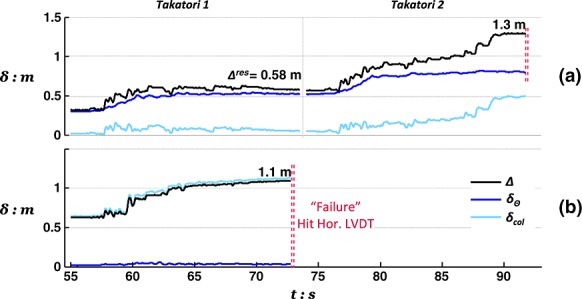
Drift response of (a) the rocking pier and (b) the conventional pier to base excitation with two successive Takatori records following the shaking sequence of Earthquake Scenario B.

**Figure 16:**
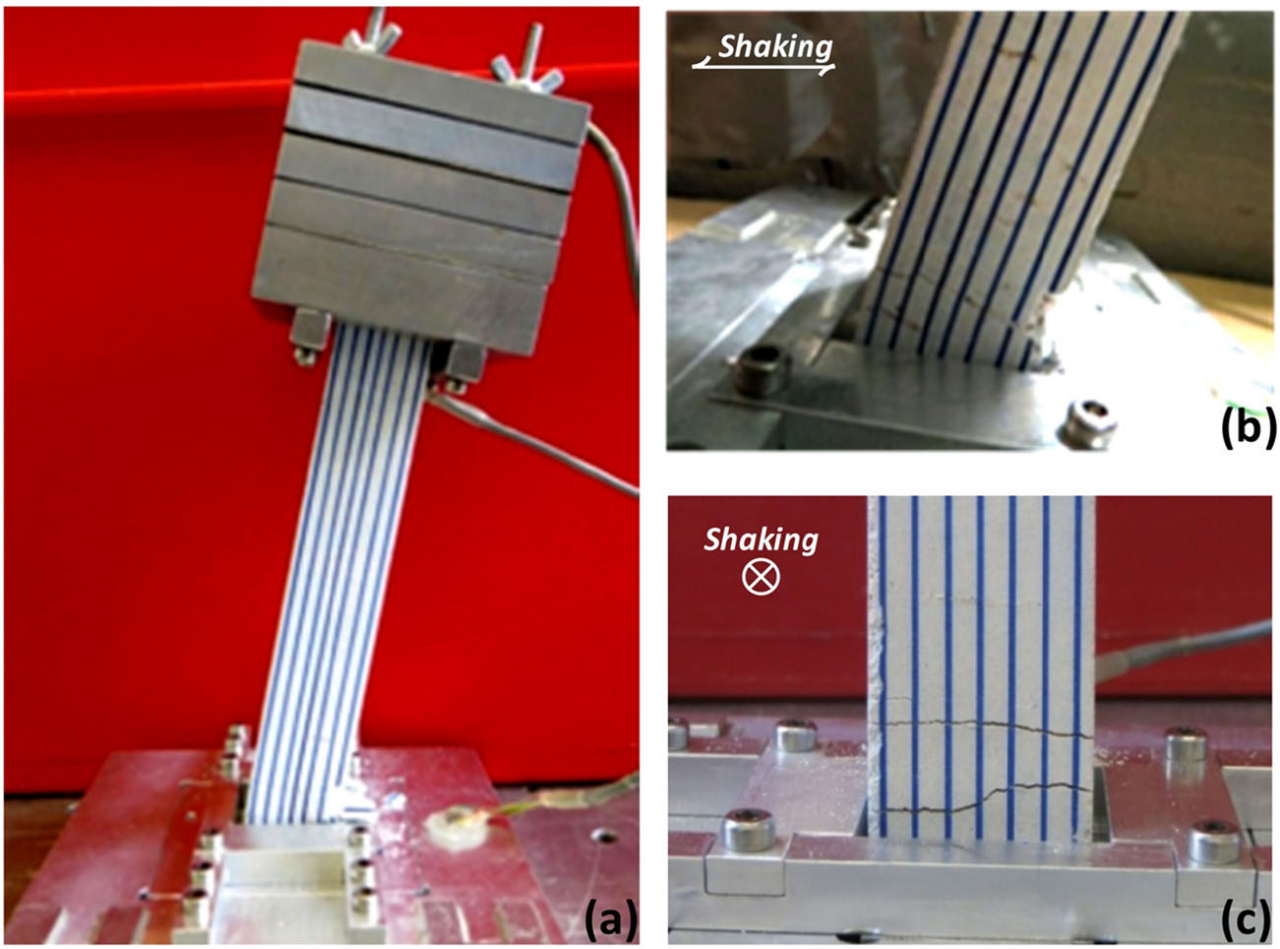
Photos of the conventional pier after Test 6 indicating the damage induced by shaking with Earthquake Scenario B followed by one additional very strong motion (the Takatori record): (a) view of the entire pier model (b) in-plane and (c) out-of-plane views of the column–foundation joint.

Because both piers survived Earthquake Scenario B without collapse, testing was continued by applying the deleterious Takatori motion. Figure 15 shows the recorded displacements at the deck of each pier. Demonstrating surprising resistance, the rocking pier survives this extreme shaking reaching a permanent deck drift due to rotation (rather than pier flexure) of 58 cm (note that this value is lower than the total drift of the conventional pier after the preceding shaking events). In fact, the second shaking with the Takatori excitation was required to induce failure of the rocking pier. In contrast, the conventional pier failed just after the application of the first Takatori motion. Figure 16 shows images of the conventional pier model after testing, showing the significant structural damage at the base of its RC column, in this case, in the plane of shaking. Again, the damage pattern (hinge length, crack formation, and compressive spalling) implies realistic modeling of the actual response of RC elements.

## 5. Concluding Remarks

This experimental campaign has provided proof of the concept of deliberately designing for foundation nonlinearity to render RC structures safe under intense seismic excitation. Emphasis is placed on the response of RC bridge piers designed in accordance with modern seismic codes and hence having well-confined cross sections. In contrast to previous studies, these piers are modeled at a highly reduced scale (1:50) using a recently developed scale model reinforced concrete, which captures the behavior of prototype RC sections with a high level of fidelity. The seismic performance of a moderately tall pier supported on a square footing on top of a layer of medium-dense sand was studied through a series of dynamic centrifuge experiments. Two design alternatives are considered: (i) conventional capacity design, in which the foundation is as usual overdesigned, guiding plastic hinging into the superstructure and (ii) rocking isolation design, in which the foundation is deliberately underdesigned to promote uplifting and soil yielding, guiding plastic deformation below the ground. The performance of the two design alternatives is evaluated and compared with the emphasis on the resistance of potential plastic hinge zones to cumulative damage due to successive shaking protocols. The testing sequence involved two different shaking scenarios, representing particularly destructive earthquake motions either preceded or followed by earthquakes closer to those against which the pier was designed.

On the basis of the presented tests, the following conclusions can be drawn:

(1) The utilized small-scale concrete model simulates the behavior of a prototype RC section reliably, allowing greatly improved prediction of the detailed nonlinear seismic response of RC structures.
(2) Rocking isolation design consistently exhibits superior performance compared with conventional capacity design, irrespective of the tested shaking scenario. Nonlinear response of the soil footing interface essentially acts as an effective and resilient energy dissipation ‘fuse’ showing substantially increased ductility capacity and resistance against cumulative damage despite the successive and intense seismic shaking. In both shaking scenarios, the rocking-isolated pier survived the excessive demands imposed by particularly strong shaking sequences, which caused catastrophic structural failure of the conventionally designed pier.
(3) Counterintuitively, the rocking-isolated pier was found to be advantageous also in terms of drift demands suffering comparatively lower deck displacements in all of the studied loading cases.
(4) Exhibiting what is known as a ‘sinking response’, the rocking foundation was found to accumulate significant settlement, this being identified as the only drawback of the rocking isolation design. Naturally, owing to its significantly lower *FS_V_*, the rocking foundation is prone to suffering increased settlements in comparison with the overdesigned foundations involved in conventional capacity design. The effect of such settlements on performance of the bridge could not be measured in the presented tests, where the continuity of the deck and the interaction between consecutive piers have not been taken into account. Yet, it should be noted that settlements are expected to be significantly limited should the pier be founded on a denser soil stratum (*D_r_* > 60%), dense enough to actually support the option of a shallow foundation or be subjected to a less deleterious shaking. Moreover, this drawback may be remediated through soil improvement measures. In fact, previous experimental studies on rigid oscillators 52 have shown that improvement, that is, densification of a soil layer of depth equal to the foundation width (4 m deep in this particular case) may drastically reduce rocking-induced foundation settlements, thereby alleviating concerns about the idea of implementing rocking isolation in practice.
